# Toward a microbial Neolithic revolution in buildings

**DOI:** 10.1186/s40168-016-0157-2

**Published:** 2016-03-29

**Authors:** David S. Thaler

**Affiliations:** Biozentrum, University of Basel, Klingelbergstrasse 50/70, CH - 4056 Basel, Switzerland

**Keywords:** Microbiome, Buildings, Built environment, Neolithic revolution, Extracellular DNA, Hygiene hypothesis, Biodiversity hypothesis, NGS

## Abstract

The Neolithic revolution—the transition of our species from hunter and gatherer to cultivator—began approximately 14,000 years ago and is essentially complete for macroscopic food. Humans remain largely pre-Neolithic in our relationship with microbes but starting with the gut we continue our hundred-year project of approaching the ability to assess and cultivate benign microbiomes in our bodies. Buildings are analogous to the body and it is time to ask what it means to cultivate benign microbiomes in our built environment. A critical distinction is that we have not found, or invented, niches in buildings where healthful microbial metabolism occurs and/or could be cultivated. Key events affecting the health and healthfulness of buildings such as a hurricane leading to a flood or a burst pipe occur only rarely and unpredictably. The cause may be transient but the effects can be long lasting and, e.g., for moisture damage, cumulative. Non-invasive “building tomography” could find moisture and “sentinel microbes” could record the integral of transient growth. “Seed” microbes are metabolically inert cells able to grow when conditions allow. All microbes and their residue present actinic molecules including immunological epitopes (molecular shapes). The fascinating hygiene and microbial biodiversity hypotheses propose that a healthy immune system requires exposure to a set of microbial epitopes that is rich in diversity. A particular conjecture is that measures of the richness of diversity derived from microbiome next-generation sequencing (NGS) can be mechanistically coupled to—rather than merely correlated with some measures of—human health. These hypotheses and conjectures inspire workers and funders but an alternative is also consequent to the first Neolithic revolution: That the genetic uniformity of contemporary foods may also decrease human exposure to molecular biodiversity in a heath-relevant manner. Understanding the consequences—including the unintended consequences of the first Neolithic revolution—will inform and help us benignly implement the second—the microbial—Neolithic revolution.

## Background

This article is situated in the context of efforts to encourage creative interdisciplinary collaborations among architects, building engineers, chemists, immunologists, epidemiologists, physicians, and microbiologists. This review and commentary was stimulated by the author’s participation in the Sloan symposium: Healthy buildings 2015-Europe whose summary in this special issue of the journal microbiome contains the telling statement “There was general consensus that while the applied microbiology developments emerging in this research community—first and foremost, DNA recovery methodology and in particular, next-generation sequencing—have had notable impacts as judged by common academic metrics; however, these advances have not successfully translated into paths which are available for practitioners to apply such methods or interpret these results with confidence in the field.” [1]. Despite the courteous language of scholarship as well as grammatical imprecision, the message is clear: Attaining relevance for this incipient and promising field is not assured. By hypothesis, our highest probability path to attaining relevance requires identifying and occasionally suggesting extensions and alternatives to currently favored ideas and approaches. Contradictions in this article are not about matters of fact; they arise from considering alternative ideas for how microbiome research can contribute to understanding and enhancing the built environment’s effects on human health. Tables 1 and 2 tend toward pedagogy; they are given in the spirit of friendly interdisciplinary invitation. Microbiome, microbiotia, and related terms in this document refer to “little itty bitty living things,” i.e., inclusive of eubacteria, archea, fungi, protists, and cells of multicellular differentiated organisms (animals and plants in common usage) that are present in the environment detached from the main body; it is also inclusive of all phage and viruses.

**Table 1:** The analogy of a building to a human body and roles of the microbiome in each

Doors as the mouth	Humans are a major source of indoor microbes. Contagion of pathogens is well documented. The transfer of a health-promoting microbiome through buildings is plausible but not demonstrated [73], i.e., the possibility of healthful analogs of “Typhoid Mary” [74].
Outer surfaces of a building and human skin	The barriers between inside and outside are semipermeable and somewhat selective. So far, the microbiome studies of buildings have focused on interior spaces. External surfaces and interstices of buildings are a source of interior microbial presence [11].
Lungs as the HVAC system, windows, and walls (especially pre-energy efficiency walls)	HVAC systems are notorious in cases of pathogen growth and dispersal. They are also candidates for monitoring and perhaps cultivation of a benign microbiome rather than attempt sterile systems constantly at risk of dangerous inoculation. HVAC, doors, and windows are sites of intended and unintended exchange with the outer environment. Windows allow unfiltered access to outdoor air. This may be a key to the prevention of asthma in farm environments, i.e., there may be a benefit from microbial diversity originating from active microbes outside, but metabolically inert inside, the building.
Plumbing as the digestive system and excretory system	The inside of wastewater plumbing may be coated in biofilm. Any presumption of complete isolation of this wastewater microbiota from occupants merits re-evaluation. Analogous to the discussions of human inoculation at birth, a building’s wastewater system is a candidate for pre-occupant deliberate inoculation with a benign microflora. We do not at this time know what such an inoculation would consist of, how stable it would turn out to be, and what its consequences would be during normal function and during times of stress such as sewage overflow or burst pipes.
The nervous system as thermostats, alarms, and smart networked buildings	The information on metabolism of buildings is currently limited. For microbes, the single most important information would be monitoring of moisture in many—including hidden such as wall interior—locations. Sentinel microbes could complement electronic measures. Human occupants and their choices (e.g., whether to open a window and ventilate, where to set the thermostat) participate in a building’s nervous system!

**Table 2:** Background for architects and building engineers: basic concepts of the hygiene, microbial biodiversity (inclusive of bacteria, fungi, protists, and viruses), and food epitope hypotheses

Interdisciplinary work—in this case, between architects, building engineers, and microbiologists—requires extra effort for clear communication. This table contains some background for the hygiene-microbial biodiversity hypothesis and the food epitope alternative proposed in the text. A caveat: “When you teach you are lying all the time. Of course at advanced levels you are lying a lot less but you are still lying all the time.” [75].	
Specific molecules have specific shapes and specific molecules recognize each other by their complementary shapes. The fitting together of complementary shapes is analogous to a lock and its key. Specificity based on matching complementary molecular shapes is a foundational and central idea in molecular biology [76].	
Many but not all molecular recognition epitopes are based on proteins. Proteins are comprised of combinations of 20 amino acids. The specific shape of a protein molecule depends on its amino acid sequence as specified by the DNA sequence of its gene. In this context, a different allele of the same gene has a slightly different DNA sequence that encodes a slightly different amino acid sequence that in turn leads to a protein that is slightly different [77]. For example, the same antibody might recognize a protein encoded by a different allele but the binding may have subtly altered kinetics.	
The part of a molecule that is complementary to another molecule is called an epitope. Molecules specifically recognize each other if, and only if, they have complementary epitopes. Most human proteins are encoded by the DNA sequence of genes received from the gametes of the parents but immune system proteins are different: they are encoded by new alleles of immune system genes that continue to be generated in adult life. (Non-protein epitopes and variation in their shapes are of great biological interest and importance but are less understood and even harder to explain.)	
Antibody-encoding and T cell receptor (immune system) genes are selected by the epitopes they are exposed to. The number and type of different epitopes that the immune system is exposed to has important and only partially understood consequences for the entire organism’s resistance to infection and probability of autoimmune disease. The hygiene hypothesis and biodiversity hypotheses propose that exposure to a diverse set of microbial epitopes aids healthy immune system development and function.	
An apt metaphor for the hygiene and biodiversity hypotheses is found in the poetic lines “A lot of people don’t have much food on their table/But they got a lot of forks’n’ knives/And they gotta cut somethin” [78]. The relevance of this poetic metaphor is the specific hypothesis that if the immune system does not experience a diverse and appropriate set of epitopes from microbes and/or food, then it is more likely to inappropriately target epitopes of self and thereby predispose to autoimmune and hypersensitivity syndromes.	
The consensus sequence is a single sequence and encodes a single epitope. In a population of genes, some have a slightly different sequence, these are called different alleles of the same gene, and some encode slightly different proteins with different epitopes. The population of alleles forms a “cloud” or “quasispecies” around the consensus sequence.	
In the food epitope hypothesis, the role that the hygiene or biodiversity hypotheses assign to microbial epitope diversity is partially assigned to food epitope diversity in the food is in turn a function of allelic diversity in the food crops.	
Different uses of the word “epitope” can lead to confusion: (a) “Near epitopes” differ in small ways that allow them to be bound to the same antibodies and/or T or B cell receptors but with different kinetics and (b) “far epitopes” which are different parts of the antigen. If the antigen is a protein, “near epitopes” might represent adjacent and near-adjacent amino acids, whereas “far epitopes” would be distinct peptides that can be completely separated and shown to bind to independently with minimum cross-reactivity. Most immunological literature does not distinguish very well between “near” and “far” epitopes. An authors’ meaning has to be derived from usage. Examples of “far” epitopes are found in the characterization of a stereotyped set of epitopes characterized by neonatal antibodies [79], as well as omics surveys of the antibody [80] and T/B cell receptor repertoires [81]. The meaning of the word “near” epitope is evident in papers on viral quasispecies and immune evasion [82]. The food epitope hypothesis is that consuming a population of “near” epitopes in food promotes development and maintenance of a more healthful immune system.	

### Précis

1. Humans have actively cultured plants and animals for over 10,000 years and have received benefits from such endeavors (Neolithic revolution). Important progress is currently being made in the century-long project of understanding and culturing benign metabolically active gut microbiomes.
2. The analogy of buildings and the human body suggest a potential for benign microbiomes in buildings. Related ideas proposed here include (a) building tomography for the non-invasive detection of moisture, (b) sentinel microbes, (c) seed microbes on moisture-vulnerable internal surfaces, and (d) seed biofilms in outflow plumbing.
3. Actionable wisdom for building practitioners (architects, building engineers, remediation specialists) has not followed from DNA recovery and next-generation sequencing (NGS) in contrast to the still-essential contributions of classical microbiology. One bottleneck in NGS relevance is that current applications do not differentiate among meanings of the term “microbiome.” These distinctions offer a route to relevance and appear technically challenging but within reach.
4. The hygiene and microbial biodiversity hypotheses have merged with NGS to suggest that microbial sequence diversity provides a measure of health. This idea is both ingenious and inspiring, but it may be wrong. Aspects of hygiene-biodiversity hypotheses are examined: (a) A portion of unique sequence found in microbiome NGS studies may never have existed in living cells. (b) There may be not-yet-found keys to simplify today’s apparently irreducible complexity. (c) Non-microbial sources of epitope diversity may complement and perhaps supersede the relative contribution of changes in microbial diversity to human health.
5. An unintended consequence of the first Neolithic revolution makes it likely that humans are exposed to less epitope variation in food. By hypothesis, uniformity of foodborne epitopes may also contribute to vulnerabilities in health. The quantitative and qualitative distinctions and interactions of food and microbial epitope exposure merit study.

### The microbial Neolithic revolution

The Neolithic revolution—when our species transitioned from hunter and gatherer to domesticator and cultivator—is almost complete with respect to macroscopic food and can now become science-based stewardship [2]. In contrast, humans remain hunter-gatherers with respect to the microbial world in which our species is embedded. Our microbiomes have no doubt changed due to changes in civilization (e.g., agriculture and urbanization) but this has been unintentional and is in contrast to the deliberate ways that hunting and gathering have transformed into a deliberate and specific macroscopic agriculture of specified plants and animals. The beginnings of more deliberate microbial Neolithic transition are underway with respect to bodily, especially the gut, microbiomes. However, regarding the external and internal surfaces of our buildings and our clothes, humans remain hunter-gatherers and often attempted microbial genocidists [3]. The problem, and the opportunity, is that we do not live in a sterile world. Just as nature abhors a vacuum, the living world abhors sterility. To the extent we succeed in sterilizing them, surfaces and substances are uniquely available for opportunistic microbes. Biocidal agents themselves can be directly or indirectly hazardous to human health [4] sometimes in surprising ways such as promoting tolerance to and the evolution against clinical antibiotics [5]. As one alternative or complementary approach, we can search for and create opportunities for introducing and possibly even cultivating benign microbiota in our built environments. We must also be honest critics and skeptics about the nature of the unknowns and the possibility of unintended consequences. Our gut microflora has become the exemplar against which other hypotheses of health-promoting microbiomes may be compared. Prebiotics and specific inoculation to optimize the gut microflora are now clinically relevant though not yet widely practiced [6], an idea that has been around with varying degrees of acceptance and success for over a hundred years [7].

### Analogies of microbiomes in buildings and bodies

Buildings can be analogized to bodies and bodily microbiomes analogized to microbiomes in the built environment. Where do the ideas fit and where do they fail? Table 1 elaborates the analogy of a human body to a building [8] and candidate analogies of microbiomes in each. Future possibilities for deliberate inoculation in the built environment include surfaces such as the exterior and interior of walls, pipes, textiles such as carpets, furniture, and clothes. Moist and wet surfaces including pipes especially for outflow are candidates for deliberate cultivation of a benign and helpful microflora.

A weak point of this analogy is the apparently different role played by active microbial metabolism and growth. The fact of periodic defecation is proof that microbes in the gut actively metabolize and grow. Renewal proves growth and growth proves metabolism. There is an abundant, rapidly growing, and important literature (that will not be reviewed here) that strongly implies that “the right” gut microbiome contributes to healthful development and function of the organism. In contrast, there are no definitive health-positive examples of active metabolism and growth of microbes in buildings (other than that in or on the occupants).

### Defining a microbiome

The word “microbiome” is routinely used to describe several distinct entities (Table 3). Conflation of microbiome types limits the value that can be gained from interpretation of sequence data. The microbiome research community is making an effort to standardize protocols for DNA extraction and purification as well as the NGS processing pipeline. Unfortunately, the currently recommended protocols [9] do not distinguish types of microbiomes (Table 3). Methods appear available (Table 3 legend) but are not widely validated or applied. Approaches that distinguish among the metabolically active, the potentially active, dead, and extracellular DNA would likely enhance the relevance of NGS to all aspects of microbial ecology and microbiome analysis. Until microbiome types are distinguished in NGS analyses, practical workers concerned with identifying sick buildings and performing biological remediation will probably remain wise to favor classical microbiological and microbial ecology approaches [10, 11]. The hygiene hypothesis is discussed in a subsequent section but a connection here is self evident to biologists but may not be to architects and building engineers: The metabolic state of microbes determines which of the microbial compounds and immunological epitopes encoded by the DNA and RNA sequences of bacteria, fungi, protists, or human cells are actually synthesized.

**Table 3:** Distinct types of “microbiome”

Microbiome type	Characteristics
(a) Microbial ecosystem	Active metabolism with or without growth
(b) Seed microbiome	Metabolically inert but can “wake up”
(c) Dead microbiome	Epitopes and chemicals, irreversibly inert
(d) Extracellular intact DNA	Intact sequence from the once-alive
(e) Never-living recovered sequence	Pre-mutagenically lesioned DNA

The word “microbiome” currently conflates several categories that can be considered as distinct but related: (*a*) A “microbial ecosystem” is an actively metabolizing and growing microbial community, the intestinal microbiome being the exemplar. (*b*) A “seed” microbiome. Consider the relationship of the seed rack at a garden store to the fields in which plants are growing. Many microbes (inclusive of viruses, bacteria, and micro-eukaryotes such as fungi) in the dry state remain viable and able to grow when conditions allow. (*c*) The “dead sequence” microbiome from irreversibly non-viable cells, spores, eukaryotes, and viruses. In one study, the ratio of total to colony-forming fungal spores was 100:1 in indoor samples [83]. (*d*) Extracellular DNA. Approximately half of the microbial DNA in soil is extracellular [84, 85]. (*e*) Extracellular DNA is chemically stable but not informationally unchangeable. Without the enzymatic repair processes present in living cells, premutagenic lesions accumulate due to physical-chemical processes such as heat, light, ionizing radiation, and oxidation [86]. When lesioned DNA is a template for polymerase, novel sequences are generated by a variety of mechanisms. 8-Oxoguanine, the most common product of reactive oxygen damage, leads predominantly to G > T transversions [87]. Deamination of cytosine leads after a couple of rounds of replication to C > T mutations [88]. Abasic sites and chain breaks can lead to bridging PCR which creates new sequences as copy-choice assemblages of templates in the original sample [89–91]. By the hypothesis proposed here, some of the sequences seen only once in NGS studies [92, 93] were never present in even a single living cell and in fact were never present as an intact sequence but are consequent to reading premutagenic lesions on damaged and largely extracellular templates. This possibility could be tested by exhaustive DNase treatment followed by heat inactivation of the DNase prior to normal extraction protocols. More than 90 % of the microbial DNA sequences reported from nature have never been cultivated, [94] and a large fraction of sequences seen only once have not been proven to have ever existed inside a living microbe. Some of this unique sequence may be an artifact consequent to recovering damaged DNA. The prediction is that a fraction of the “seen only once” sequence will disappear with prior DNase treatment. Treatment with DNase plus proteinase K is reported as able to distinguish between DNA that is present in live cells from cells that are dead or extracellular DNA [95]. Extracellular DNA is vulnerable to DNase treatment alone [96]. RNA analysis also has potential to differentially note growing cells in “omics” style sampling but with caveats. In *Escherichia coli*, the proportion of rRNA to rRNA-encoding genes increases as a linear function of growth rate [97]. This intriguing property whose functional basis remains uncertain [98] is not universal and it is unsure how general the effect is. Vibrio has a much smaller change in rRNA as a function of growth rate [99]. As speculation, more ribosomes in stationary phase may allow cells to enter rapid growth with less lag phase. ATP analysis is used as a marker of bacterial viability and activity [100], but again, there are caveats because ATP is sometimes quite stable [101]. One only has to recall PCR reaction conditions to note the stability of triphosphate nucleotides through multiple cycles of heating and cooling

Intermediate cases: Some microbes are able to undergo repeated cycles of wetting and drying without specialized forms such as spores. Desiccation tolerance can be a property either of individual cells [102] or of microbial communities [103]. Viable but not culturable (VBNC) microbes are a class based on physiological state first characterized and named by Rita Colwell in the context of aquatic *Vibrio cholera* [104]. VNBC cells are not spores but require specific conditions to revive. Once revived growth and metabolism are normal, natural transformation can resurrect “dead” DNA and is an important mechanism of horizontal gene transfer [105, 106]. An indeterminate fraction of the microbiome sequence in, e.g., household dust is either “seed,” “dead,” extracellular DNA, or generated sequence

### Growing health-promoting microbes in buildings

Health-positive roles for a metabolically active building microbiome may be awaiting our discovery or invention. Candidates worth investigating include the following: (a) Actinomycetes are ubiquitous in building walls [12]. With their complex and varied secondary metabolism, actinomycetes have long been the source of novel compounds including antibiotics and antifungals [13]. There is good evidence that some species of actinomycetes create toxic products that can interact synergistically with fungi to the detriment of occupant health [14, 15]. By hypothesis, there may also exist species and strains whose metabolic products inhibit fungi associated with building-related symptoms (BRS—more popularly known as sick building syndrome, SBS) and/or specific diseases that can be transmitted via the built environment. (b) Biofilms and planktonic growth often occur in plumbing and HVAC systems. These may provide an opportunity for intentional and knowledge-based cultivation that at least occupies the niche where random inoculation occasionally leads to niche occupation by microbial pathogens [16–18]. In infants, the first inoculated microbiome has long-term effects [19]. By hypothesis, the same will prove true for water outflow pipes. The relevant time scale of buildings can be short, e.g., mold can grow in a few days after a flood but the scale includes years, decades, and in some cases centuries. The longer time scales differ from laboratory experiments and the usual ecological contexts, e.g., soil and fecal, that because they are familiar, tend to provide the mental frame for thinking about microbiology in the built environment but in the context of geomicrobiology, doubling times of centuries or even millennia have been estimated [20].

Water is a fundamental limiting factor for microbial growth. The moisture content and distribution in buildings is complex in both space and time [21]. Non-invasive tomographic quantification of moisture in buildings could become a breakthrough technology in locating microbial growth. It is proposed here that the microwave absorption and reflection properties of water [22, 23] could be used to map moisture throughout buildings including spaces (e.g., inside walls) currently denied to non-invasive methods.

How robust do we want or need the built environment to be against the sequelae of rare but intense bursts of moisture? Occurrences of intense rainfall and flooding are expected to become more frequent consequent to climate change [24]. Pipes leak and burst unexpectedly. What can be done against rare but highly consequential events? Plumbing biofilms might be seeded with microbes anticipated to benignly bias microbial sequelae. Preparations of normally dry regions might include pre-treatment inoculation of buildings (including the interior of walls) with benign microbes in a quiescent state but able to germinate and take over a niche that when wetted would otherwise be vulnerable to the growth of noxious microbes. These are hopeful speculations but they suggest research to identify desiccation-tolerant and quickly revivable benign microbes. Microbial viability through multiple cycles of wetting and desiccation appears to be a marvelous topic at the fundamental level [25, 26] whose deeper understanding could also lead to practical consequences [27]. The interactions of microbes, moisture cycles, and the moisture retention properties of building materials could become a rich area for interdisciplinary study.

Sentinel microbes that are themselves innocuous but chosen or engineered to be easy to measure could be deliberately inoculated as monitors for microbial growth allowing quantification of accumulated stable isozymes of indicators such as β-galactosidase or GFP. In a similar way, sentinel microbes could be added to foods as a way to quantify if and how much the food experienced conditions allowing microbial growth. Extensions of NGS have the potential to identify metabolically active microbes (Table 3 legend). Sentinel microbes could be developed as another window to understanding the places and conditions in buildings that promote or permit microbial metabolism.

### Hygiene and biodiversity hypotheses

The hygiene [28] and the related (microbial) biodiversity hypotheses [29] propose that diverse microbial exposure is key to the optimum development and function of the immune system (Table 2). A diversity of immune epitopes in the microbial environment is proposed to channel the immune system such that autoimmune reactivity becomes less likely. Despite—indeed because of—the attractiveness of these ideas, they should be critically examined and alternatives considered. We lack knowledge of which logical and operational definitions of biodiversity [30, 31] are most relevant to human health. Over 50 years ago, Dubos et al. demonstrated that a benign intestinal microflora protects against microbial pathogens [32]. However, proving a role for microbes is not the same as proving that microbial complexity or diversity are required—or even helpful—to do the job.

An astonishing finding contradicts a key predication of the biodiversity hypotheses regarding the role of a complex microbiome in normal intestinal development: Infection by a single norovirus strain corrects all defects associated with axenic intestinal development in the mouse [33]! There remains a great deal of suggestive and intriguing correlative but not definitive evidence in favor of the hygiene and biodiversity hypotheses [10, 34–41]. The propensity to develop asthma correlated with a low diversity of fungi in dust samples [42] and the intestinal microbiome may play a role immune conditioning regarding susceptibility to asthma [43, 44]. Gammaproteobacterial complexity on the forearm is associated with benign immune tolerance as shown by a decrease in atopy [45]. Hanski et al. favor a causal relationship in which the microbiota leads to immune tolerance while acknowledging that they cannot rule out the causally inverse interpretation that an intolerant immune reaction alters the microbiota. Subsequent studies show an immune-moderating role associated with endotoxin of one of the identified bacteria: *Acinetobacter lwoffi* in both human cells and a mouse sensitization protocol [46] but immune tolerance is a double-edged sword. Other experimental and clinical contexts show bacterial endotoxin-triggering immune responses that are in some experimental systems protective against infection but in others harmful to the organism [47]. Immunological tolerance can predispose to disease—susceptibility to infectious disease—as well as to health as in the diminishment of atopy. Tolerance toward environmental mycobacteria built-up as a consequence of birth and early life in environments rich in this biota may be part of the reason it is hard to make an effective vaccine against *Mycobacterium tuberculosis* and induced tolerance during infection may also be a part of the *M. tuberculosis* strategy for pathogenesis [48].

### Is sequence complexity the medium or the message?

The hypothesis that microbiome diversity measured by NGS of genes encoding small subunit RNA (ssuRNA) is a positive driver of a building’s or an individual’s microbiome health [49] deserves consideration but, based on current evidence, not acceptance. Despite the cleverness and attractiveness of the idea, ecosystem and evolutionary complexity do not scale with measures of stability, “useful”, or “adaptive” [50, 51]. “Just so stories” in the popular press or TED talks may imply that differences in sequence distribution reflect microbial adaptation or benign “appropriateness” to a niche but correlation is not causation. Microbial distribution in what might have been thought to be reasonably uniformly mixed oceans appears largely consequent to the seeding of microorganisms that differ only in neutral mutations [52, 53]. The situation may be even more arbitrary and coincidental in the context of buildings. Sequences in a sample of dry dust from a building may sometimes (often?) represent nothing more than vagaries of air and human traffic circulation rather than microbial adaptation to the niche in which they are found. And yet, the health consequences of microbial epitope-human immune system interactions may be profound even if chance brought them together. Complexity in sequencing data as well as in the potential interactions of microbes with an already complicated indoor chemistry [54] are facts but complexity in interpretation can also be consequent to a deficit in theory [55]. Random sampling alone might explain why “complex” and “rich in diversity” microbiomes are more likely to contain rare but specific actinic compounds. In science, one should stay alert to the possibility that apparent complexity represents an intermediate state. Simplicity at the core of complexity remains a reasonable null hypothesis. On the other hand, the current state of knowledge cannot rule out complexity itself as a possible answer. Molecular mechanism(s) by which microbiome complexity itself might promote host health have been alluded to in an almost romantic fashion that is inspiring but not well articulated in forms that are testable and falsifiable experimentally. A meta-analysis finds that children raised on farms have approximately 25 % lower asthma prevalence [56] and one explanation is an ameliorative effect of exposure to a diverse microbiota [37]. Attempts are made to rule out confounding factors, but alternatives to the microbial biodiversity hypothesis include genetic predispositions, exercise, altered diet, environmental pollution either outdoors or indoors, changes in sleep patterns, and vitamin D insufficiency [57].

### The food epitope hypothesis

**Fig. 1:**
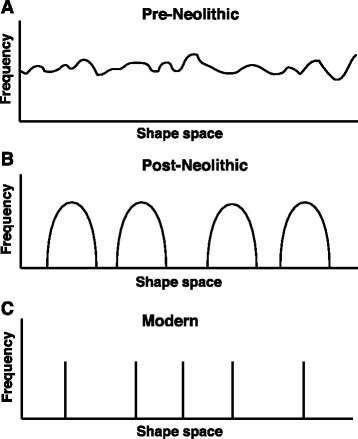
The food epitope hypothesis. Epitope space [68] of food consumed by humans before (**a**), after (**b**) the Neolithic revolution, and the modern phase of agricultural genetics (**c**). All parameters of shape space are compressed into the *X*-axis. The *Y*-axis is a frequency distribution. **a** In the pre-Neolithic phase of our species, we were hunter-gatherers. The food that we ate was maximally (for our species) dispersed in the living world and we were exposed to more dietary epitopes. The variation in epitopes of each food source was based on the allelic variance within plant and animal populations [69]. Each food source was population based containing the genetic and allelic diversity that implies [70]. **b** The Neolithic revolution, i.e., the advent of agriculture and domestication of both plant and animal food sources. The epitope classes and their dietary distribution became relatively restricted. Most previous food sources were no longer consumed but a few made up the majority of the diet. Within these food sources, the amount of variation was also constrained because even in early agriculture, artificial selection limits the allelic and by implication the epitope diversity in food [71]. **c** The modern agricultural era of the last ca. 50 years has led to human food becoming more genetically uniform within each species of plants and livestock [72]. The associated distribution of food epitopes in shape space has become punctate

Diet has been discussed as a way to alter immune function by several mechanisms including modification of the host commensal microbiome [58–61] but proposed here is an additional mechanism by which epitope diversity in the food itself influences the immune system: The Neolithic revolution brought about a decrease in the variety of foods consumed by our species [62]. Breeding practices in agriculture have changed over the last 50 years and it is a reasonable proposition—but remains to be proven—that consequently the allelic diversity of major foodstuffs destined for human consumption has been further reduced. Genetic uniformity of crops in contemporary agriculture is discussed in the context of food security and the susceptibility of plant and animal agriculture to infectious disease [63, 64]. The hypothesis proposed here is that there are also immunological, developmental, and neuro-psychiatric [65, 66] consequences of a punctate distribution of dietary food-derived near epitopes (Fig. 1 and for a definition of “near” epitopes, see the last paragraph of Table 2). The hypotheses for health benefits of exposure to a rich microbial diversity appear also applicable to the allelic diversity of food. The two views are not qualitatively at odds since immune function can be jointly modulated by diet and microbial exposure [67]. Quantitatively, daily food consumption with its associated epitopes exceeds exposure to microbial epitopes by orders of magnitude. Thus, we return at the end of this review and commentary to the point on which we began. The first Neolithic revolution has been our species’ most important innovation and has also led to large modifications of earth’s biosphere. Deepening our understanding of the first Neolithic revolutions consequences—including unintended consequences—will inform opportunities for further benign developments. Our species may not have another 10,000 years to get the second Neolithic revolution- the microbial Neolithic revolution- right.

## Conclusions

The microbial Neolithic revolution is underway with regard to the gut microbiome but its extension to buildings requires the clarification of key matters: (1) Active microbial metabolism in buildings is known to be associated and causative of SBS/BRS. Focused research will be required to learn if active microbial metabolism is in some cases, or could ever become, healthful in buildings. (2) The association of some measures of microbial diversity in buildings with some measures of human occupant health is intriguing but the responsible mechanism(s) remain unknown. Diversity may simply represent a bigger sampling more likely to contain a few (mostly unknown) key compounds or, alternatively, exposure to diversity itself may be healthful, again through mechanism(s) requiring clarification through focused research. (3) More research is needed to critically compare, contrast, hierarchically organize, quantify, and understand the health-relevant consequences of human exposure to diversity from multiple sources including microbes as well as the products of the first Neolithic revolution, food.

## Abbreviations

BRS: building-related symptoms
BRI: building-related illness
GFP: green fluorescent protein
HVAC: heating ventilation and air-conditioning
NGS: next-generation sequencing
SBS: sick building syndrome
ssuRNA: small subunit RNA (inclusive of 16sRNA in prokaryotes and 18sRNA in eukaryotic cytoplasm)
